# Inflammation-Driven Secretion Potential Is Upregulated in Osteoarthritic Fibroblast-Like Synoviocytes

**DOI:** 10.3390/ijms231911817

**Published:** 2022-10-05

**Authors:** Jakub Chwastek, Marta Kędziora, Małgorzata Borczyk, Michał Korostyński, Katarzyna Starowicz

**Affiliations:** 1 Department of Neurochemistry, Maj Institute of Pharmacology Polish Academy of Sciences, 31-343 Krakow, Poland; 2 Laboratory of Pharmacogenomics, Department of Molecular Pharmacology, Maj Institute of Pharmacology Polish Academy of Sciences, 31-343 Krakow, Poland

**Keywords:** osteoarthritis, fibroblast-like synoviocytes, inflammation, chemokines, RNA-seq

## Abstract

Osteoarthritis (OA) is one of the most common joint pathologies and a major cause of disability among the population of developed countries. It manifests as a gradual degeneration of the cartilage and subchondral part of the bone, leading to joint damage. Recent studies indicate that not only the cells that make up the articular cartilage but also the synoviocytes, which build the membrane surrounding the joint, contribute to the development of OA. Therefore, the aim of the study was to determine the response to inflammatory factors of osteoarthritic synoviocytes and to identify proteins secreted by them that may influence the progression of OA. This study demonstrated that fibroblast-like synoviocytes of OA patients (FLS-OA) respond more strongly to pro-inflammatory stimulation than cells obtained from control patients (FLS). These changes were observed at the transcriptome level and subsequently confirmed by protein analysis. FLS-OA stimulated by pro-inflammatory factors [such as lipopolysaccharide (LPS) and tumor necrosis factor alpha (TNFα) were shown to secrete significantly more chemokines (CXCL6, CXCL10, and CXCL16) and growth factors [angiopoietin-like protein 1 (ANGPTL1), fibroblast growth factor 5 (FGF5), and insulin-like growth factor 2 (IGF2)] than control cells. Moreover, the translation of proteolytic enzymes [matrix metalloprotease 3 (MMP3), cathepsin K (CTSK), and cathepsin S (CTSS)] by FLS-OA is increased under inflammatory conditions. Our data indicate that the FLS of OA patients are functionally altered, resulting in an enhanced response to the presence of pro-inflammatory factors in the environment, manifested by the increased production of the previously mentioned proteins, which may promote further disease progression.

## 1. Introduction

Osteoarthritis (OA) is one of the most common joint pathologies; it causes pain and disability in more than 10% of the population of developed countries [1]. The direct causes of this disease are still unknown, but the main risk factors are female gender, obesity, and, most importantly, age [1]. OA is becoming an increasingly common problem in an aging society, so it is essential to investigate new and more effective therapies to prevent its development based on a thorough understanding of its causes. Aging is a major risk factor in the development of OA, and this may be due to long-term exposure to joint strain and mechanical damage [2]. An equally important issue is to identify markers that signal the early stages of OA, allowing treatment to be started sooner and improving the effectiveness of the applied therapy.

The main histopathological sign of OA is degeneration of cartilage tissue, which leads to pain and limits the patient’s mobility. Closer examination, however, has revealed that the subchondral bone is also damaged in advanced OA, making the joint progressively less resistant to loading [3]. In the early stages of OA, macroscopic changes of the joint are not observed in patients, but ECM degeneration occurs with the involvement of proteases. In the late stages of the disease, extensive degeneration of the cartilage and subchondral part of the bone is observed, leading to a severe pain response and reduced patient mobility. Additionally, in the advanced stage of OA, synovitis is observed, characterized by excessive hypertrophy of the synovium [4], which is the membrane surrounding the joint that is responsible for synovial fluid production, cartilage nutrition, and lubrication. It is made up of two types of cells (called synoviocytes): type A, which are cells with a phenotype resembling macrophages/monocytes and type B, which are fibroblast-like synoviocytes (FLS) [5]. During the progression of OA, particularly in the knee, macroscopic changes in the structure of the synovium—an increase in its volume and vascularisation—are observed. These signs are indicative of the development of synovitis [6]. It can be described as a chronic inflammatory condition that is observed even in the early stages of OA [7], at which time a mixture of chemokines, cytokines, and proteolytic enzymes is secreted into the synovial fluid, resulting in increased immune cell activity and reorganization of the extracellular matrix of cartilage tissue [8]. The factors listed earlier may contribute to the progression of OA and are responsible for cartilage degradation, bone remodeling, osteophyte formation, joint inflammation, and loss of normal joint function [8]. The OARSI (Osteoarthritis Research Society International) emphasizes inflammation as an important factor in disease development, defining OA as “a disorder involving movable joints characterized by cell stress and extracellular matrix degradation initiated by micro- and macro-injury that activates maladaptive repair responses including pro-inflammatory pathways of innate immunity” [9].

Maintaining joint balance is a complex process that involves all joint building tissues; disruption of this state leads to the development of several diseases, including OA, such as rheumatoid arthritis [10], gouty arthritis [11] or Juvenile idiopathic arthritis [12]. In its physiological state, synovial fluid is responsible for adequate lubrication of the joint, but it also nourishes the tissues, drains waste products, and carries enzymes, growth factors, and cytokines, enabling the joint to function efficiently [13]. Metalloproteases are secreted in healthy joints primarily by chondrocytes and allow for controlled extracellular matrix reorganization [14]. Synoviocytes are the main source of lubrication molecules, such as hyaluronan and proteoglycan 4 [15]. However, during the development of OA, the secretory potential of all the joint tissues changes, leading to an increase in extracellular factors in the synovial fluid such as cytokines (IL1α, IL18, and TNFα), chemokines (CCL2, CXCL10 and CXCL12) [16] and the gradual onset of joint degeneration symptoms. The most characteristic symptom of OA is a degradation of articular cartilage which consists mostly of dense extracellular matrix (ECM), produced by chondrocytes, which make up only about 1–3% of this tissue [17]. Due to the structure of the joint cartilage, its repair and regeneration are significantly hampered. Cartilage degeneration occurs through increased proteolytic enzyme activity on the one hand, and excessive osteogenesis, which promotes osteophytes formation, on the other [18]. Activation of these processes occurs, among other things, through the presence in the environment (synovial fluid) of factors such as cytokines, chemokines and growth factors, which may be produced by a variety of cell types, including macrophages, chondrocytes and synovial fibroblasts [8]. The changes observed in the phenotype of the synovial membrane of OA patients, as well as in the secretory potential of FLS-OA, indicate that the development of the disease may be regulated by this tissue.

To examine alterations in synovial membrane-building cells under chronic inflammatory conditions, primary cultures of FLS isolated from OA and control patients were used. To induce an immune response, cells were stimulated with two pro-inflammatory factors—lipopolysaccharide (LPS) and tumor necrosis factor alpha (TNFα). LPS, isolated from the outer membrane of Escherichia coli, leads to strong pro-inflammatory stimulation, so it is often used to model the general immune response [19,20,21,22]. TNFα occurs naturally during the development of inflammation in the human body and is produced mainly by immune cells; thus, it invokes a more physiological FLS inflammatory response [23]. Moreover, elevated levels of this factor are observed in the synovial fluid of OA patients [7,24,25]. This approach allowed us to obtain more convincing data regarding the response of FLS to inflammation and compare the pro-inflammatory effects of a substance widely used for modeling inflammation (LPS) with a more physiological cytokine (TNFα). The use of in vitro culture allowed us to observe changes in synoviocytes during the early stages of inflammation, which was investigated using cell transcriptome analysis and confirmed at the protein level using the enzyme linked immunosorbent assay (ELISA) method. Our data enabled us to identify proteins that may help researchers better understand the process of OA development and serve as markers of early changes within joints in the future or as handle points for future therapeutic strategies.

## 2. Results

### 2.1. Comparison of the Gene Expression Response to Pro-Inflammatory Stimulation between OA Patients and Healthy Donors

**Figure 1:**
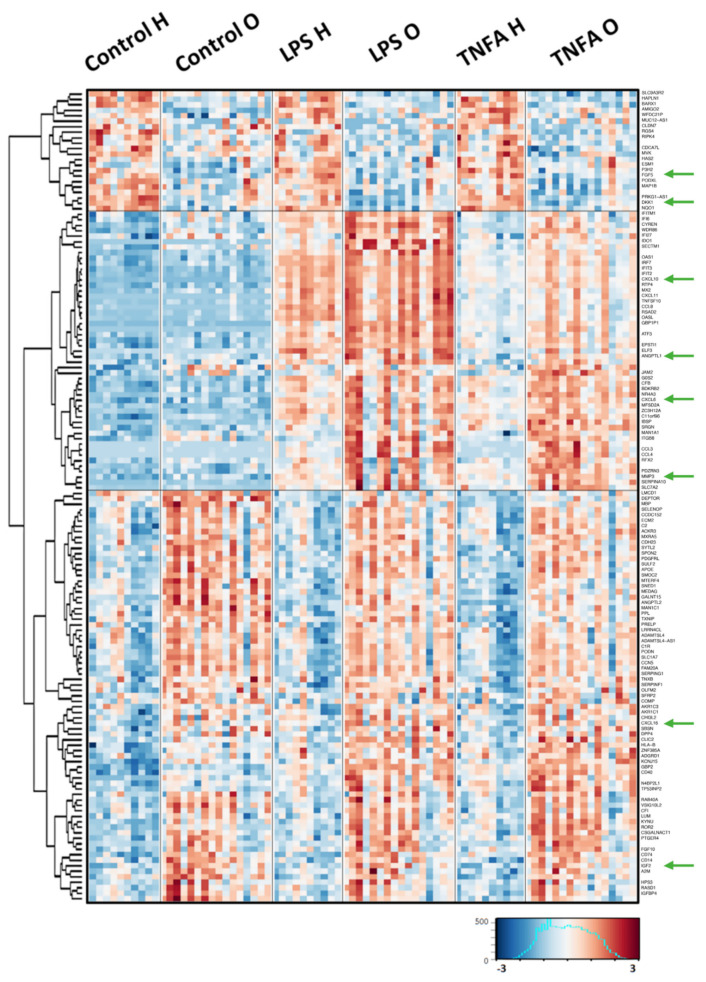
Gene expression profiling of the response to pro-inflammatory stimulation. HFLS cells from healthy donors (H) and OA patients (O) were stimulated with either LPS (LPS) or TNFα (TNFA) for four hours, and their transcriptomes were sequenced. RNAseq results are shown as a heatmap and include transcripts with a genome-wide significance from three-way ANOVA for the stimulation factor (FDR-corrected *p* < 0.05) and additional custom filters to select genes with differences between the H and OA groups (FDR-corrected *p* disease < 0.3, fold change tnf.h. vs. tnf.o > 1). This filtering selected 148 genes. The intensity of the colored rectangles represents transcript abundance levels. The presented level is proportional to the row z-score values (between −3 and 3), as displayed on the bar below the heatmap image. The blue values correspond to the samples with the abundance below the mean, and red values to the samples with the higher abundance. Hierarchical clustering was performed using correlation as a distance measure. The full list of differentially expressed transcripts is presented in Supplementary Table S1. Group naming: Control H—healthy unstimulated group, O—OA unstimulated group, LPS H—cell from healthy controls stimulated with LPS, LPS O—OA cells stimulated with LPS, TNFA H—healthy cells stimulated with TNFα, TNFA O—OA cells stimulated with TNFA.

Human fibroblast-like synoviocytes (HFLS) cells from healthy donors and OA patients were stimulated with either LPS or TNFα, and their transcriptomes were sequenced. RNA sequencing results were analyzed with three-way repeated measures ANOVA on log2-transformed and normalized (FPKM) gene counts. The obtained results are available in Supplementary Table S1. Both factors influenced the expression of numerous genes. There were 2741 genes differentially expressed according to the disease factor (FDR < 10%, 1243 upregulated, 1498 downregulated), while 7169 genes were significantly regulated by stimulation (FDR < 10%, 3804 upregulated, 3365 downregulated). Eight transcripts showed a significant interaction between the disease and stimulation ANOVA factors. As thousands of differentially expressed genes were detected, for the heatmap, the results were filtered with a custom filter (FDR < 5% for the stimulation factor, FDR < 30% for the disease factor, log2 fold change with TNFa stimulation >1) to identify genes differentially expressed between OA and healthy subjects additionally affected by the stimulation by TNFα. This filtering yielded 148 genes (Figure 1). There were three main regulation patterns of those genes visible as clusters on the heatmap: (i) genes downregulated in the cells from patients under both the control and stimulated conditions; (ii) genes upregulated in patient cells under stimulated conditions but not under control conditions; and (iii) genes upregulated in patient cells under all conditions. Enrichment analysis was performed, drawing on the Bioplanet and GO Molecular Function databases (Supplementary Table S2). In the GO Molecular Function, 11 pathways were significantly enriched, with adjusted *p*-values < 0.05 and at least 3 genes in the pathway. The top three terms were: chemokine activity (GO:0008009, 7 genes), chemokine receptor binding (GO:0042379, 7 genes) and oxidoreductase activity, acting on NAD(P)H, quinone or similar compound as acceptor (GO:0016655). In the Bioplanet database we found 27 pathways fulfilling the same criteria. The top three pathways were TGF-beta regulation of extracellular matrix (26 genes), Interferon alpha/beta signaling (11 genes), Interleukin-1 regulation of extracellular matrix (13 genes).

### 2.2. Signaling Protein Secretion Analysis Using the ELISA Method

The results obtained in the transcriptome sequencing experiment were then confirmed using the ELISA method. Proteins for analysis were selected based on significant differences in mRNA expression between stimulated HFLS and osteoarthritic human fibroblast-like synoviocytes (HFLS-OA) cells; additionally, only secretory proteins were considered according to the UniProt database. This was done because OA affects all tissues of the joint, proteins secreted by HFLS during inflammation appear to impact disease progression. Due to the drastic increase in the factors released into the environment by HFLS during inflammation, as observed by other researchers [26,27,28], it has been decided to analyze the proteins that are up-regulated in the used model, as they seem to have the significant influence on the regulation of processes involved in joint degeneration. Eight of the twelve proteins selected for further analysis are shown on the heatmap (Figure 1, green arrows). The other chosen factors (CTSK, CTSS, retinoic acid receptor responder protein 1 and 2 (RARRES1, RARRES2)) were selected based on the analysis of Supplementary Table S1. Additionally, the literature review showed that RARRES2 is elevated in synovial fluid [29] and RARRES1 [30] in multiple tissue samples of OA patients. The expression of the other two proteins (CTSK and CTSS) was investigated due to increased protease activity in the development of OA.

Preliminary analyses using the ELISA method demonstrated that proteins, such as chemokines (CXCL6, CXCL10, and CXCL16) and growth factors (ANGPTL1, FGF5, and IGF2), are secreted into the medium by HFLS. However, we did not detect in media the presence of proteolytic enzymes (MMP3, CTSK, and CTSS) or other proteins (RARRES1, RARRES2, and Dickkopf WNT Signaling Pathway Inhibitor 1 (DKK1)), whose coding mRNAs differed between the study groups, so they were analyzed in cell lysates.

#### 2.2.1. Chemokine Secretion during Inflammation Is Upregulated in HFLS-OA

**Figure 2:**
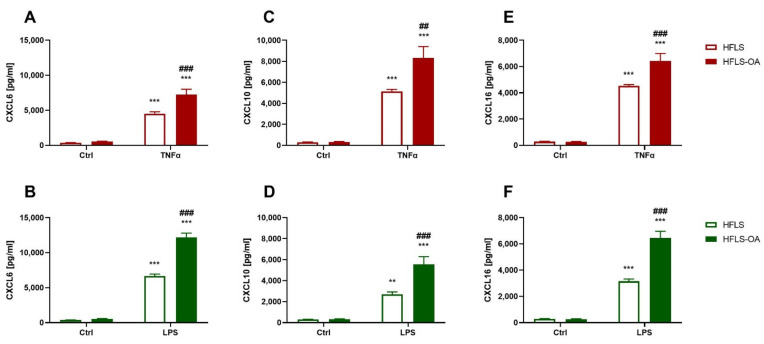
Chemokine secretion levels by HFLS and HFLS-OA stimulated with TNFα or LPS measured using the ELISA method. Upregulation of chemokine level secretion measured in media using the ELISA method. Cells were stimulated for 24 h with 10 ng/mL of TNFα (**A**,**C**,**E**) or LPS (**B**,**D**,**F**). Afterward, the media were collected, and CXCL6 (**A**,**B**), CXCL10 (**C**,**D**), and CXCL16 (**E**,**F**) levels were determined. Data are presented as pg/mL ± SEM and analyzed with two-way ANOVA followed by Tukey’s post hoc test. ** 0.01 > *p* > 0.001; *** *p* < 0.001 vs. control group. ## 0.01 > *p* > 0.001; ### *p* < 0.001 HFLS vs. HFLS-OA stimulated groups. HFLS, human fibroblast-like synoviocytes; HFLS-OA, osteoarthritic human fibroblast-like synoviocytes; TNFα, tumor necrosis factor alpha; LPS, lipopolysaccharide; ELISA, enzyme linked immunosorbent assay; CXCL6, chemokine (C-X-C motif) ligand 6; CXCL10, chemokine (C-X-C motif) ligand 10; CXCL16, chemokine (C-X-C motif) ligand 16.

Since OA is usually accompanied by synovitis, the secretion of three chemokines (CXCL6, CXCL10, and CXCL16) into the culture medium by HFLS and HFLS-OA was determined. We observed that both of the pro-inflammatory stimulants we used significantly elevated the level of chemokines released by the cells compared to the controls (Figure 2A–F). Moreover, stimulated OA cells secreted significantly more of each chemokine than the control group cells (Figure 2A–F).

#### 2.2.2. HFLS-OA Released More Growth Factors after Pro-Inflammatory Stimulation

**Figure 3:**
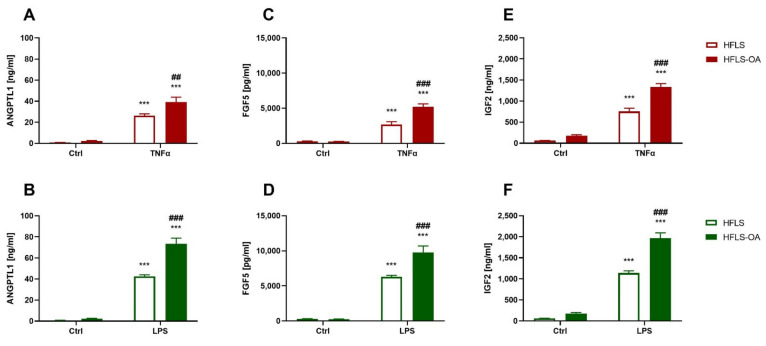
Growth factor secretion levels by HFLS and HFLS-OA stimulated with TNFα or LPS measured using the ELISA method. Secretion of growth factors measured in media using the ELISA method. Cells were stimulated for 24 h with 10 ng/mL of TNFα (**A**,**C**,**E**) or LPS (**B**,**D**,**F**). Afterward, the media were collected, and ANGPTL1 (**A**,**B**), FGF5 (**C**,**D**), and IGF2 (**E**,**F**) levels were determined. Data are presented as pg or ng/mL ± SEM and analyzed with two-way ANOVA followed by Tukey’s post hoc test. *** *p* < 0.001 vs. control group. ## 0.01 > *p* > 0.001; ### *p* < 0.001 HFLS vs. HFLS-OA stimulated groups. HFLS, human fibroblast-like synoviocytes; HFLS-OA, osteoarthritic human fibroblast-like synoviocytes; TNFα, tumor necrosis factor alpha; LPS, lipopolysaccharide; ELISA, enzyme linked immunosorbent assay; ANGPTL1, angiopoietin like 1; FGF5, fibroblast growth factor 5; IGF2, insulin growth factor 2.

The second group of analyzed proteins consisted of growth factors that may be involved in joint tissue reorganization. In all stimulated groups, the selected protein (ANGPTL1, FGF5, and IGF2) secretion levels were elevated; the secretion levels measured in stimulated HFLS-OA were significantly higher than in HFLS (Figure 3A–F).

#### 2.2.3. Elevated Proteolytic Enzyme Translation in Pro-Inflammatory-Stimulated HFLS-OA

**Figure 4:**
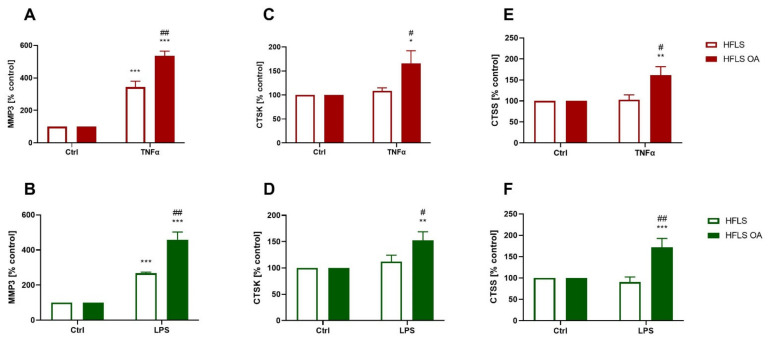
Proteolytic enzymes expression levels by HFLS and HFLS-OA stimulated with TNFα or LPS measured using the ELISA method. Expression of proteases measured in cellular lysates using the ELISA method. Cells were stimulated for 24 h with 10 ng/mL of TNFα (**A**,**C**,**E**) or LPS (**B**,**D**,**F**). Afterward, the proteins were isolated, and MMP3 (**A**,**B**), CTSK (**C**,**D**), and CTSS (**E**,**F**) levels were determined. Data were calculated as % of control ± SEM and analyzed with two-way ANOVA followed by Tukey’s post hoc test. * *p* < 0.05; ** 0.01 > *p* > 0.001; *** *p* < 0.001 vs. control group. # *p* < 0.05; ## 0.01 > *p* > 0.001; vs. HFLS-OA stimulated groups. HFLS, human fibroblast-like synoviocytes; HFLS-OA, osteoarthritic human fibroblast-like synoviocytes; TNFα, tumor necrosis factor alpha; LPS, lipopolysaccharide; ELISA, enzyme linked immunosorbent assay; MMP3, matrix metalloprotease 3; CTSK, cathepsin K; CTSS, cathepsin S.

Proteases are enzymes that are responsible for, inter alia, extracellular matrix degeneration; they are crucial for joint tissue remodeling but may also cause OA to progress. We found that pro-inflammatory stimulation caused an increase in MMP3 expression in the cellular lysates of both cell types, and the level of this protein was higher in stimulated HFLS-OA than in control cells (Figure 4A,B). Expression of cathepsins K and S was elevated only in HFLS-OA stimulated with TNFα (Figure 4C,E) and LPS (Figure 4D,F), and it was significantly higher when compared with both the control and stimulated HFLS groups.

#### 2.2.4. Changes in RARRES1, RARRES2, and DKK1 Expression in HFLS-OA during Inflammation

**Figure 5:**
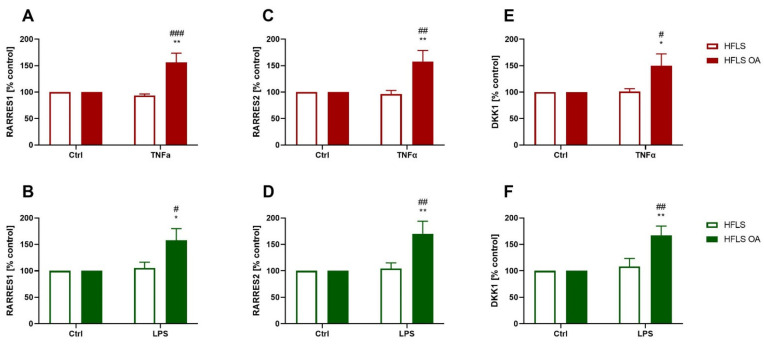
RARRES1, RARRES2, and DKK1 expression levels by HFLS and HFLS-OA stimulated with TNFα or LPS measured using the ELISA method. Expression of proteases measured in cellular lysates using the ELISA method. Cells were stimulated for 24 h with 10 ng/mL of TNFα (**A**,**C**,**E**) or LPS (**B**,**D**,**F**). Afterward, the proteins were isolated, and RARRES1 (**A**,**B**), RARRES1 (**C**,**D**), and DKK1 (**E**,**F**) levels were determined. Data were calculated as % of control ± SEM and analyzed with two-way ANOVA followed by Tukey’s post hoc test. * *p* < 0.05; ** 0.01 > *p* > 0.001; vs. control group. # *p* < 0.05; ## 0.01 > *p* > 0.001; ### *p* < 0.001 HFLS vs. HFLS-OA stimulated groups. HFLS, human fibroblast-like synoviocytes; HFLS-OA, osteoarthritic human fibroblast-like synoviocytes; TNFα, tumor necrosis factor alpha; LPS, lipopolysaccharide; ELISA, enzyme linked immunosorbent assay; RARRES1, retinoic acid receptor responder 1; RARRES2, retinoic acid receptor responder 2; DKK1, dickkopf WNT signaling pathway inhibitor 1.

The last proteins that were studied did not fit into any of the selected groups and may be involved in the regulation of various processes, such as apoptosis (RARRES1) [31], metabolism (RARRES2) [32], or cell differentiation by the Wnt signaling pathway (DKK1). Expression levels increased only in HFLS-OA treated with TNFα or LPS for every researched protein (Figure 5A–F).

## 3. Discussion

Synovitis is a well-described phenomenon and one of the main causes of the development of rheumatoid arthritis, but it has been observed that this process is involved in OA development as well [33]. Our studies have demonstrated that prolonged inflammation affecting HFLS during OA progression leads to permanent functional changes in these cells, manifested by an enhanced response to pro-inflammatory stimulation. We observed that HFLS isolated from patients with OA expressed significantly more proteins, which may regulate the immune response and cell metabolism more than in control cells.

Analysis of transcriptome changes induced by pro-inflammatory factors was performed shortly after the addition of the compounds (four hours) to observe an early inflammatory response induced by LPS or TNFα. After a longer stimulation time, a secondary cellular response may have occurred due to a cocktail of secreted substances [34], which may mask the effect of the originally applied pro-inflammatory factors. Under these conditions, the level of HFLS activation could be extremely high, making the differences between HFLS and HFLS-OA undetectable. Our preliminary studies have shown that after 24 h, the levels of secreted IL6 and CCL2 increase manyfold (Supplementary Figure S3), which may lead to secondary pro-inflammatory stimulation. The enrichment analysis carried out showed that the selected 148 genes, whose expression differs between stimulated osteoarthritic and control cells, encode proteins involved in processes such as chemokine activity, TGF-beta regulation of extracellular matrix or growth factor activity, among others. These processes are involved in the progression of OA, as described in the literature [35,36,37] and their action is based on secretory proteins. This study focused on this group of proteins because the role of HFLS and their secretory potential appears to be important in the development of OA. Some of the factors (MMP3, CTSK, CTSS, DKK1, RARRES1, RARRES2) were not determined in the cell medium, so their presence in cell lysates was examined. This may be due to the relatively low expression levels of these proteins in the used model and, in addition, the dilution of these proteins in the medium made their detection difficult. To the best of our knowledge, this is the first study based on transcriptome changes after short-term stimulation with pro-inflammatory factors in HFLS isolated from OA patients. After analysis of the data, potentially secreted factors were selected, and changes at the protein level were confirmed.

Ongoing inflammation is observed during the development of OA [8]; therefore, changes in the expression levels of proteins that regulate this process might be suitable markers of OA progression. Such factors include chemokines, which are chemoattractants for both immune and endothelial cells responsible for inflammation progression and angiogenesis. Data in the literature provide strong evidence for the involvement of chemokines in the development of OA [35]; however, these findings mainly concern the effects of these proteins on cartilage tissue or their levels in synovial fluid [38]. The RNA sequencing experiment described herein indicates that HFLS stimulation with pro-inflammatory factors upregulates the expression of more than 30 chemokines in both HFLS and HFLS-OA cells. Moreover, the enrichment analysis showed that 6 of the selected 148 genes, whose expression differs between stimulated osteoarthritic and control cells, encode chemokines, such as CCL3; CCL4; CXCL6; CXCL10; CXCL11; CXCL16 (Supplementary Table S2). Elevated levels of CCL3 are detected in the plasma of OA patients [39]; furthermore, overexpression of CCL3 and CCL4 has been detected in pro-inflammation-stimulated chondrocytes [40]. Other researchers have demonstrated chemokine expression from the (C-X-C motif) ligand (CXCL) family in rheumatoid fibroblast-like synoviocytes (CXCL6 [41]; CXCL10 [42]; CXCL11 [43]; CXCL16 [44]). Based on this result, we chose three of them (CXCL6, CXCL10, and CXCL16, which are also known as granulocyte chemotactic protein 2 (GCP-2), interferon gamma-induced protein 10 (IP-10), and phosphatidylserine and oxidized lipoprotein (SR-PSOX), respectively) whose expression was higher in HFLS-OAs than in HFLS after pro-inflammatory stimulation. Elevated expression of the CXCL6 gene was observed in OA chondrocytes [45] and FLS-rheumatoid arthritis (FLS-RA) cells [41] stimulated in vitro with IL-1β and resistin (adipokine), respectively. Moreover, upregulated secretion of CXCL16 was observed in the media from FLS-RAs and FLS-OAs stimulated with peptidoglycan [46]. Similar data were reported about CXCL10 in FLS-RAs stimulated by TNFα [47,48] and in OA chondrocytes [45]. Additionally, CXCL10 protein levels are elevated in the articular cartilage and serum of OA patients [49]; however, dermal fibroblasts stimulated with this chemokine produced significantly higher levels of hyaluronic acid and type I collagen [50]. Another potentially beneficial effect of CXCL10 is the recruitment of subchondral progenitors, which take part in the cartilage-healing process [51]. Upregulation of CXCL16 levels was observed in HFLS isolated from OA and RA patients. This chemokine stimulated the expression of receptor activator of nuclear factor κB ligand (RANKL), which promoted bone erosion [44]. The data cited above demonstrate that increased chemokine levels in the tissues of OA and RA patients may reflect an enhanced immune response and the activation of signaling pathways aimed at reconstructing damaged tissues. Both processes might serve as targets for potential therapy; however, the beneficial application of such potential therapy requires additional research.

The data presented in this study indicate that HFLS from OA patients secrete elevated quantities of growth factors after pro-inflammatory stimulation compared to control cells. Growth factors regulate processes responsible for tissue development, such as cell proliferation or differentiation, and thus, they may influence processes accompanying the development of OA. IGF-2 acts through both insulin-like growth factor 1 receptor 1 (IGF-1R) and insulin receptor A (IR-A) and is involved, inter alia, in fetal skeletal system development [52]. It has been shown that pro-inflammation-stimulated synoviocytes can secrete vascular endothelial growth factor (VEGF) [53], which may be associated with the progression of OA, by stimulating such processes as cartilage degeneration, osteophyte formation, synovitis or pain [54]. This indicates that the factors released by the HFLS into the environment may have an impact on the function of other tissues that make up the joint. In vitro studies have shown that it can also stimulate bone regeneration by activating the PI3K/AKT signaling pathway in mesenchymal stem cells, leading to osteogenesis [55]. The synovial fluid IGF-2 level is elevated in OA patients [56]; in vitro studies have demonstrated that this factor enhances extracellular matrix production by IL-1β-treated human chondrocytes via inhibition of the NF-κB pathway [57]. Other studies have reported activation of the PI3K/AKT signaling pathway via IGF-1R promoted extracellular matrix production by chondrocytes [58].

Angiopoietin-like proteins (ANGPTLs) comprise a group of eight glycoproteins (ANGPTL1–ANGPTL8) that have a similar structure and are classified as angiopoietin family proteins [59]. They are widely expressed in various tissues and take part in the regulation of numerous processes, such as inflammation, metabolism, and angiogenesis [60]. Most of the information about the functions of the ANGPTL1 protein has come from cancer research, including its role not only in the inhibition of hepatocellular carcinoma proliferation and adhesion but also its anti-apoptotic action [61]. Studies have also shown that ANGPTL1 can promote bone regeneration by inhibiting osteoclast activity (manifested by increased bone resorption), which is potentially beneficial in OA therapy [59]. Another interesting function is decreasing matrix metalloprotease 9 (MMP9) expression in Kupffer cells (also known as stellate macrophages, which are localized in the liver) by exosomal ANGPTL1 [62], mainly due to upregulation of this metalloprotease in the serum of OA patients [63].

The best-studied function of the last-tested growth factor (FGF5) is its effect on the development of the long-hair phenotype of various mammals [64]. Other studies have shown that it is an oncogenic factor in several human cancer types; it promotes cell proliferation and migration and inhibits apoptotic cell death [65,66,67]. FGF5 may also stimulate the formation of connective tissue and lead to fibrosis [68], which is observed in OA and results in joint stiffness [69]. The growth factors investigated in our study demonstrate the paracrine interaction of HFLS with other tissues. In addition, the secreted proteins may regulate the entire joint’s response to inflammation.

The third group of proteins examined in this study were proteolytic enzymes, whose main extracellular function is reorganization of bone and cartilage structures. An established balance between catabolic and anabolic processes is essential for proper joint function. However, during the development of OA, protease overactivation was observed, resulting in erosion of cartilage and subchondral bone [70]. Our data have shown that pro-inflammatory-stimulated HFLS-OAs expressed more MMP3, CTSK, and CTSS than control cells, but cathepsin levels were similar in the stimulated HFLS cells and the control group cells. Numerous studies have demonstrated increased levels of metalloproteases in OA progression, and this group of enzymes is one of the most important factors for cartilage decomposition [71]. One metalloprotease is MMP3, whose overexpression was observed in OA patients’ cartilage [72], synovial tissue [73], and synovial fluid [74]. Due to the presence of this protein in OA patients’ joint tissues, it has been proposed as one of the biomarkers of disease progression [75].

Cathepsins are cysteine proteases that are active in low pH, so they usually function inside lysosomes. CTSK is involved in the degradation of types I and II collagen and aggrecan, which form the extracellular matrix of chondrocytes. Overexpression of the CTSK protein was observed in cartilage and synovial tissue isolated from patients with OA [76]. An inhibitor of this enzyme (balicatib) was shown to inhibit the development of OA, but the study was discontinued because of side effects [77]. Moreover, in Ctsk(-/-) knockout mice with OA induced by joint instability, disease progression was significantly delayed, while the expression levels of other proteases (such as MMP13 and ADAMTS5) decreased compared to the control group [78]. CTSS is a lysosomal protease that digests damaged or unwanted proteins inside lysosomes, but it is also released by macrophages at the site of inflammation and can effectively hydrolyze aggrecan, leading to cartilage extracellular matrix destruction [79]. Its gene expression was elevated in synovial tissue isolated from patients with OA [80], and the protein was upregulated in the synovial fluid of RA patients [81]. Upregulation of CTSS might be beneficial in inflamed synovial tissue because of its anti-fibrotic properties [82], especially as synovial fibrosis is an important factor in causing joint stiffness [83]. The changes in the expression of proteolytic enzymes observed in our study during the inflammatory response indicate that there is increased activation of catabolic processes in HFLS cells, which may lead to the degradation of joint building tissues, as well as to the prevention of synovial fibrosis or the reorganization of the tissue structure.

In the last group of proteins tested, increased expression was observed only in pro-inflammatory-stimulated cells isolated from OA patients. DKK1 appears to be a critical factor in regulating joint regeneration. This protein is an inhibitor of the Wnt signaling pathway, which modulates differentiation, proliferation, and cell death [84]. Its level in synovial fluid is positively correlated with the severity of OA [85]. DKK1 secretion was increased in synovial fluid and synoviocytes isolated from RA patients. Moreover, FLS-RAs stimulated by pro-inflammatory factors overexpressed this protein [86]. Weng et al. showed that DKK1 promotes OA chondrocyte apoptosis, which may lead to cartilage deterioration [87]. Other authors have observed that inhibition of DKK1 in inflammatory arthritis animal models resulted in the enhancement of bone remodeling processes [88]. There are no data on the effect of RARRES1 on the development of joint disease, but it has been shown to have pro-inflammatory and fibrosis-promoting effects through activation of the NF-κB signaling pathway [89]. RARRES2 (also known as chemerin) was overexpressed in the serum of OA [90] and RA [91] patients, but it was not correlated with disease stage. RARRES2 belongs to the adipokine family, and this protein may be a link between OA and obesity (its upregulation is confirmed in both disorders) [92]. This hypothesis has not yet been confirmed; however, there are data that appear to recognize the involvement of this protein in the development of OA. RARRES2-stimulated FLS-OAs secreted elevated CCL2 levels in the culture medium [93] and inhibited the proliferation of chondrocytes isolated from OA patients [94]. Considering the above literature, the results of our experiments demonstrate that osteoarthritic synoviocytes express more of the above-mentioned proteins under pro-inflammatory stimulation than control cells. This is consistent with the other results presented in this work, that HFLS-OA produce faster and more factors that can influence OA progression.

In summary, our studies have shown that synoviocytes during OA progression undergo permanent molecular changes. These changes contribute to the increased secretion of extracellular factors, which are responsible for long-term changes in the entire synovial membrane (and thus in the synovial fluid and cartilage), which is directly affected by factors secreted into the synovial fluid. A thorough understanding of the mechanisms responsible for synovitis development and maintenance in OA is crucial for developing effective therapy. Appropriate action in the early stages of the disease encourages the hope of finding targeted therapy that could slow disease progression and thereby provide better functioning of OA-affected joints.

## 4. Materials and Methods

### 4.1. Human Fibroblast-Like Synoviocytes (HFLS) Preparation

Synovial tissue was obtained from a total of 16 patients with OA and 10 control patients (with no OA signs). The OA group included 5 males and 11 females, aged 49–87 years old, with an average age of 70 years. The OA patients underwent elective knee joint replacement surgery. The non-OA group included 9 males and 1 female, aged 18–47 years old, with an average age of 32 years; samples were collected during anterior cruciate ligament injury (ACL) reconstruction surgery under the approval of the Local Ethics Committee in Katowice (Approval numbers 2/2011 and RS/14/18). No joint disease other than mechanical damage was found in the donors.

HFLS were isolated as described before [95]. Synovial tissue samples from OA were obtained immediately after opening the knee joint capsule. A piece of synovial tissue up to 9 cm^2^ in size was dissected and divided. The control tissue (synovial membrane fragments up to 3 cm^2^) was placed in culture medium. Fragments of synovial tissue were minced and placed in culture media supplemented with 0.4 mg/mL of Liberase (Roche Diagnostics, Basel, Switzerland). Digestion was carried out for at least 1 h at 37 °C on a shaking platform. The resulting suspension was filtered (70 μm) and centrifuged at 300× *g* for 10 min. The pel-let was then treated with erythrocyte lysis buffer (20.7 g NH_4_Cl, 1.97 g NH_4_HCO_3_, 0.09 g EDTA, and 1 L H_2_O) for 5 min and recentrifuged for 10 min at 300× *g*. The pellet was resuspended in Roswell Park Memorial Institute (RPMI) 1640 (Gibco, Billings, MT, USA) with 10% fetal bovine serum (FBS) and transferred to a 75 cm^2^ tissue culture flask. After overnight incubation, cells were supplemented with a fresh medium.

### 4.2. Cell Culturing and Treatment

The cells were cultured as descrbed before [95] in RPMI 1640 supplemented with GlutaMax, 10 mM 4-(2-hydroxyethyl)-1-piperazineethanesulfonic acid (HEPES), 10% FBS and 100 U/mL penicillin, and 0.1 mg/mL streptomycin (all reagents obtained from Gibco, Billings, MT, USA) at 37 °C in a humidified atmosphere containing 5% CO_2_. The dosage of pro-inflammatory factors-LPS and TNFα (Gibco, Billings, MT, USA) was experimentally determined at 10 ng/mL for both compounds (Supplementary Figure S1A–D). Two different times of stimulation were used for RNAseq and ELISA experiments: 4 and 24 h, respectively. Times were experimentally determined with polymerase chain reaction (PCR) and ELISA method (Supplementary Figures S2A–D and S3A–D respectively). Cells between passages 2–6 were used in experiments.

### 4.3. RNA Isolation

RNA was isolated with the TRIzol reagent (cat #15596026, ThermoFischer, Waltham, MA, USA) following the manufacturer’s protocol. The total RNA concentration was measured using an ND-1000 Spectrometer (NanoDrop Technologies Inc., Wilmington, DE, USA). The quality of RNA was determined by using an RNA 6000 Nano Lab-Chip Kit and an Agilent Bioanalyzer 2100 (Agilent, Santa Clara, CA, USA). Based on the RNA integrity number (RIN > 7.5) values, 68 samples were selected for sequencing.

### 4.4. Library Preparation and Transcriptome Sequencing

A total amount of 1 μg RNA per sample was used as input material for the RNA sam-ple preparations. mRNA from eukaryotic organisms was enriched using oligo(dT) beads from NEBNext^®^ Poly(A) mRNA Magnetic Isolation Module (cat # E7490L-NEB). Subsequently, sequencing libraries were generated using NEBNext Ultra II Di-rectional RNA Library Prep Kit for Illumina^®^ (cat # E7770L-NEB, Illumina, San Diego, CA, USA) following the manufacturer’s recommendations. Briefly, fragmentation was carried out using divalent cations under elevated temperature in NEBNext First Strand Synthesis Reaction Buffer (5×). First-strand cDNA was synthesized using random hexamer primer and M-MuLV Reverse Transcriptase (RNaseH-). Second strand cDNA synthesis was subsequently performed using DNA Polymerase I and RNase H. In the reaction buffer, dNTPs with dTTP were replaced by dUTP. The remaining overhangs were converted into blunt ends via exonuclease/polymerase activities. After adenylation of 3′ ends of DNA fragments, NEBNext adaptors with hairpin loop structure were ligated to prepare for hybridization. To preferentially select cDNA fragments of 250~300 bp in length, the library fragments were purified with AMPure XP beads (cat # A63987 Beckman Coulter, Brea, CA, USA). Then 3 μL USER Enzyme (NEB) was used with size-selected, adaptor-ligated cDNA at 37 °C for 15 min followed by 5 min at 95 °C before PCR. Then PCR was performed with Phusion High-Fidelity DNA polymerase, Universal PCR primers, and Index (X) Primer. Finally, products were purified (AMPure XP beads) and library quality was assessed using the Agilent High Sensitivity DNA Kit (cat # 5067-4626) on the Agilent Bioanalyzer 2100 system (Agilent Technologies, Santa Clara, CA, USA). Sequencing was performed by the Novogene Experimental Department (Beijing, China). The clustering of the index-coded samples was performed on a cBot Cluster Generation System (cat# SY401-2015, Illumina) using TruSeq PE Cluster Kit v3-cBot-HS (cat# PE-401-3001, Illumia) according to the manufacturer’s instructions. After cluster generation, the libraries were sequenced on a NovaSeq 6000 Illumina platform using NovaSeq 6000 S2 Reagent Kit v1.5 cat. 20028314 -(300 cycles) and 150 bp paired-end reads were generated (minimum 12 Gb and 40 M).

### 4.5. RNA-Seq Data Analysis

RNA-seq analysis was performed as described previously with some modifications, described below [96]. All samples were checked for quality with fastQC v0.11.8 [97] and aligned to the human reference genome (grch38 from index provided by hisat2) with hisat2 2.1.0 [98]. Cufflinks v 2.2.1 package [99] and GTF from the Ensembl gene database (release 100) [100] were used to quantify (cuffquant) and normalize (cuffnorm) transcripts to fpkms (Fragments Per Kilobase of transcript per Million fragments mapped). All statistical analyses were performed with R software 3.6 [101]. Statistical significance was tested using 3-way RM ANOVA (between-subject factors: disease x treatment, within subject factor: patient) on log2 (1 + FPKM) values with a false discovery rate (FDR) adjustment. For the overall analysis, we assumed the standard FDR threshold of 10%, which resulted in thousands of regulated genes upon stimulation (full results are available in Supplementary Table S1); therefore, we applied further filters to choose genes to present on the heatmap. Genes with mean log2 (1 + FPKM) < 1 were excluded from the analysis. Enrichment analysis was performed with the EnrichR tool. We analyzed the Bioplanet and GO molecular function databases. Pathways were considered to be enriched if at least 3 genes from the submitted list of genes were in the pathway and the adjusted *p*-value was <0.05.

### 4.6. Protein Isolation

For protein isolation, cells were washed with ice-cold PBS, next harvested and lysed with ice-cold RIPA buffer (ThermoFischer, Waltham, MA, USA), supplemented with protease inhibitors cocktail (Halt, ThermoFischer, Waltham, MA, USA) and centrifuged at 14,000× *g* for 15 min at 4 °C. The supernatants were stored at −20 °C until further use. Protein concentration was measured with a BCA kit (ThermoFischer, Waltham, MA, USA) following the manufacturer’s protocol.

### 4.7. Enzyme-Linked Immunosorbent Assay (ELISA)

The chemokines: CXCL6 (C-X-C Motif Chemokine Ligand 6), CXCL10 (C-X-C Motif Chemokine Ligand 10), CXCL16 (C-X-C Motif Chemokine Ligand 16), and the growth factors: ANGPTL1 (Angiopoietin-related protein 1), FGF5 (Fibroblast Growth Factor 5), and IGF-2 (Insulin-like growth factor 2) were measured in supernatants harvested 24 h after LPS or TNF-α treatment. The protein levels of the CXCL6 (Human GCP2 (Granulocyte Chemotactic Protein 2) ELISA Kit, ELK Biotechnology, Wuhan, China), CXCL10 (Human IP-10/CXCL10 ELISA Kit, Elabscience, Wuhan, China), CXCL16 (Human CXCL16 ELISA Kit, Elabscience, Wuhan, China), ANGPTL1 (Human ANGPTL1 ELISA Kit, ELK Biotechnology, Wuhan, China), FGF5 (Human FGF5 ELISA Kit, ELK Biotechnology, Wuhan, China) and IGF-2 (Human IGF-2 ELISA Kit, Elabscience, Wuhan, China) were measured using commercially available enzyme-linked immunosorbent assay kits according to the manufacturers’ instructions. The detection limits were as follows: CXCL6 (15.63–1000 pg/mL), CXCL10 (7.81–500 pg/mL), CXCL16 (0.31–20 ng/mL), ANGPTL1 (0.16–10 ng/mL), FGF5 (15.63–1000 pg/mL) and IGF-2 (7.81–500 ng/mL).

The proteases (MMP3 (Matrix Metallopeptidase 3), CTSK (Cathepsin K), CTSS (Cathepsin S)), and the other proteins (RARRES1 (Retinoic Acid Receptor Responder 1), RARRES2 (Retinoic Acid Receptor Responder 2), and Dkk-1 (Dickkopf related protein 1)) were measured in lysates harvested 24 h after LPS or TNF-α treatment. The protein levels of MMP3 (Human Total MMP-3 Quantikine ELISA Kit, R&D Systems, Minneapolis, MN, USA), CTSK (Human CTSK ELISA Kit, ELK Biotechnology), CTSS (Human CTSS ELISA Kit, ELK Biotechnology), RARRES1 (Human RARRES ELISA Kit, ELK Biotechnology), RARRES2 (Human CHEM ELISA Kit, ELK Biotechnology), and Dkk-1 (Human DKK1 ELISA Kit, ELK Biotechnology) were measured using commercially available enzyme-linked immunosorbent assay kits according to the manufacturers’ instructions. The detection limits were as follows: MMP3 (0.2–10 ng/mL), CTSK (0.16–10 ng/mL), CTSS (0.16–10 ng/mL), RARRES1 (0.16–10 ng/mL), RARRES2 (0.16–10 ng/mL), and Dkk-1 (0.16–10 ng/mL).

### 4.8. Statistical Analysis

Statistical analyses were performed using Statistica 13.3 software (Statsoft, Tulsa, OK, USA). All biochemical experiments were carried out under the same conditions for all samples, regardless of the type of treatment. All data were obtained in independent experiments and are presented as the means ± SEM (standard errors of the means). All groups were compared using a factorial analysis of variance (ANOVA) with Tukey’s post hoc test for multiple comparisons, to show statistical significance with assumed *p* < 0.05.

## Data Availability

Not applicable.

## Supplementary Materials

The following supporting information can be downloaded at: https://www.mdpi.com/article/10.3390/ijms231911817/s1.
