# Supplementary material for: Inflammation-Driven Secretion Potential Is Upregulated in Osteoarthritic Fibroblast-Like Synoviocytes
Source: Int J Mol Sci. 2022 Oct 5;23(19):11817. doi: 10.3390/ijms231911817 (PMC9570304; doi:10.3390/ijms231911817)
Supplement: Supplementary file 1 [file ijms-23-11817-s001.zip › Supplementary data.pdf]

Supplementary methods

Cell culture

The cells were cultured in RPMI 1640 supplemented with GlutaMax, 10 mM HEPES, 10% FBS and 100 U/mL penicillin, and 0.1 mg/mL streptomycin (all reagents obtained from Gibco, USA) at 37°C in a humidified atmosphere containing 5% CO_2. Cells were stimulated with LPS or TNFα (Gibco, USA) in different concentrations of 1-50 ng/ml or 1-25 ng/ml respectively for 24 hours. In the second part of the experiment, cells were incubated with 10 ng/ml LPS or TNFα for different time points (1, 4, 24, and 48 hours).

Real-time PCR

RNA was isolated as mentioned in the main text.

For reverse transcription, NG dART kit (EurX, Poland) was used and the procedure was carried out as described in the manufacturer's kit instruction. Briefly, 500 ng of isolated RNA was mixed with supermix which contained reverse transcriptase, RNase inhibitor, dNTPs, and oligo(dT). Samples were incubated for 1 hour at 50°C and the reaction was terminated by heating for 5 minutes at 85°C.

For the real-time PCR reaction iTaq universal probes supermix (Bio-Rad, USA) and TaqMan probes labeled with FAM (Ccl2: Hs00234140_m1, Il6: Hs00174131_m1, B2m: Hs00187842_m1 [ThermoFischer Scientific, USA]) were used. Data were calculated with ΔΔCt method and normalized on B2m expression.

ELISA

Cells were stimulated with 10 ng/ml of LPS or TNFα and media from each group were collected at different time points (1, 4, 24, and 48 hours). Secreted IL6 or CCL2 protein were measured with an ELISA kit (88-7066 and 88-7399 respectively, ThermoFischer Scientific, USA) according to the manufacturer’s manual.

Supplementary Table S1. RNA-seq results.

Sheet 1 - results for all genes, sheet 2 - Results for selected genes.

Column labels:

gene_stable_ID, gene_symbol - gene identifiers

fdr.disease, fdr.treatment, fdr.interaction - ANOVA results p-values after FDR correction

t.test.ctrl.h.vs.o, directions.ctrl.h.vs.o - t-test p-value and direction of change compared with control. this t-test compares unstimulated cells

fold.change.tnf.h, fold.change.tnf.o, fold.change.lps.h, fold.change.lps.o, fold.change.tnf.h.vs.o, fold.change.lps.h.vs.o - fold changes in abs(log2(FPKM + 1)) format

difference.tnf,difference.lps - two additional fold changes representing the difference between response to stimulation between H and O groups in abs(log2(FPKM + 1)) format

sd - sd of the group with the highest sd of all the groups for this gene

subsequent columns have individual log2(FPKM + 1) values for all the sample, coding: H - healthy, OA - osteoarthritis group, next 2-3 digits - patient ID, last digit - stimulation (1- ctrl, 3 - tnfa, 4 - lps)

Supplementary Table S2. Enrichment analysis results for the GO Molecular Component and Bioplanet databases. All pathways with adjusted p-value < 0.05 are included in the table. Pathways with at least 3 genes are marked with distinct color.

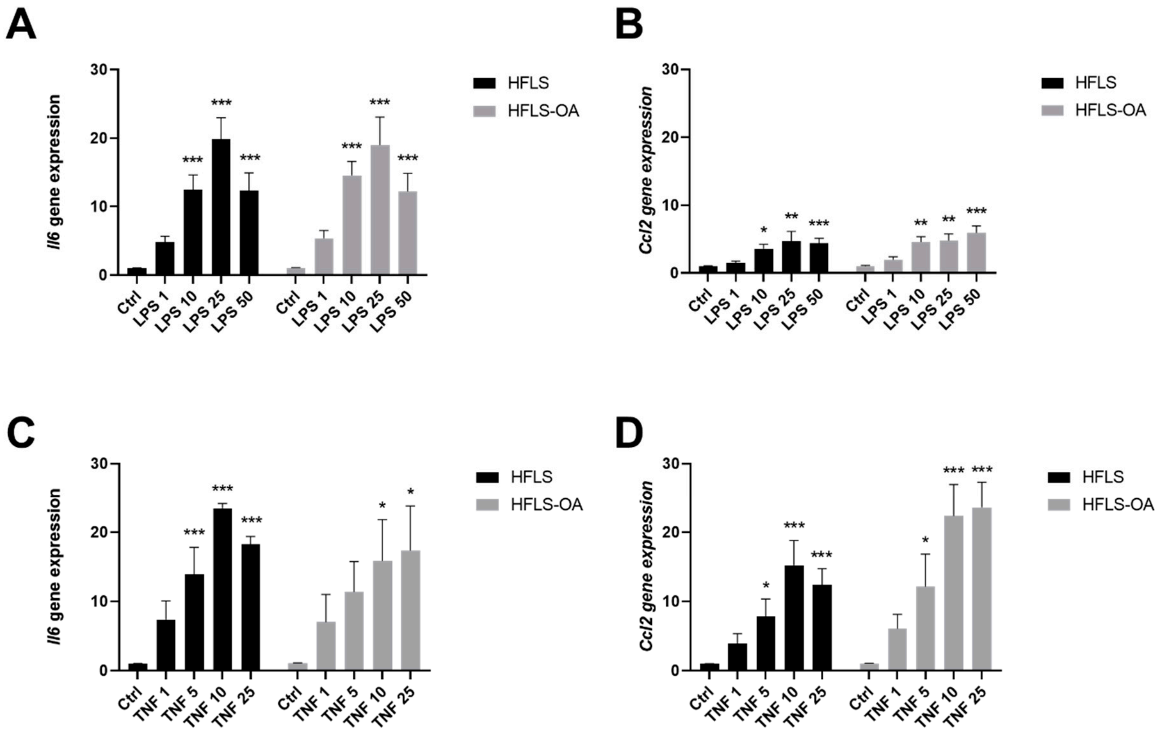

Figure S1.

HFLS and HFLS-OA cells were stimulated with different concentrations of LPS (1-50 ng/ml, (**A**,**B**)) or TNFα (1-25 ng/ml, (**C**,**D**)) for 24 hours. Ccl2 or Il6 genes expression were measured with a real-time PCR method. Data are presented as mean fold change ± SEM, and analyzed with one-way ANOVA followed by the Dunnett post hoc test. * $p < 0.05$; ** $0.01 > p > 0.001$; *** $p < 0.001$ vs. control group.

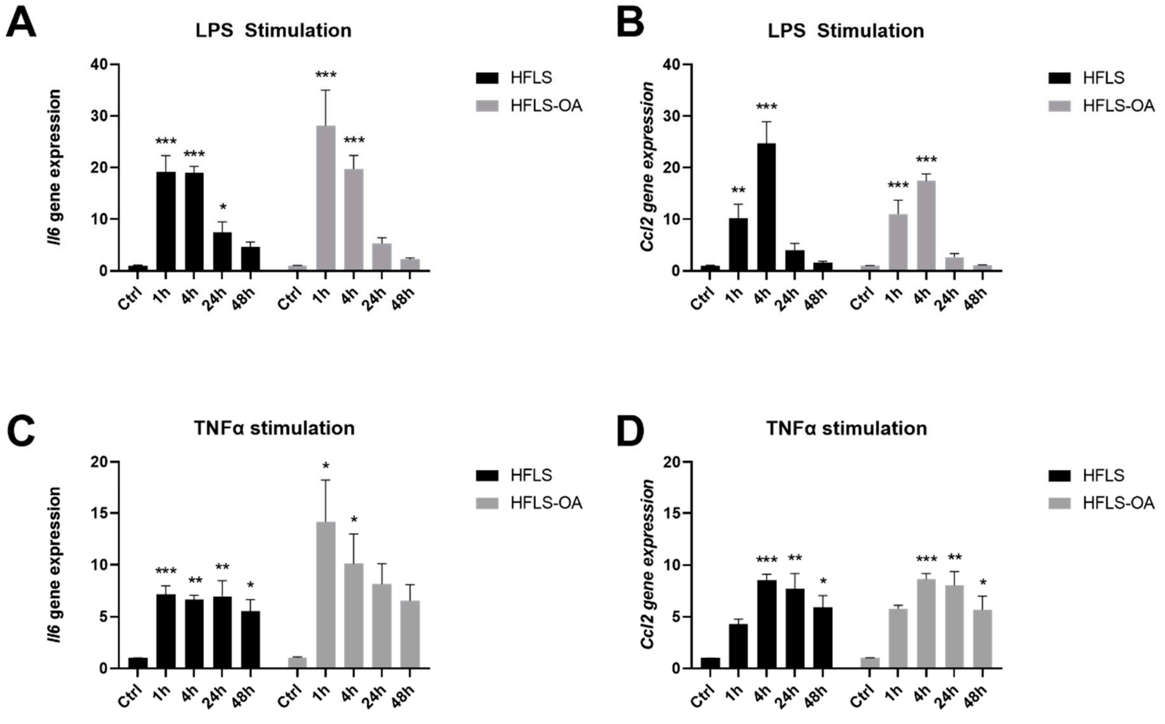

Figure S2.

HFLS and HFLS-OA cells were stimulated with 10 ng/ml LPS (**A**,**B**) or TNFα (**C**,**D**) for different time points (1, 4, 24, and 48 hours). Ccl2 or Il6 genes expression were measured with a real-time PCR method. Data are presented as mean fold change ± SEM, and analyzed with one-way ANOVA followed by the Dunnett. * $p < 0.05$; ** $0.01 > p > 0.001$; *** $p < 0.001$ vs. control group.

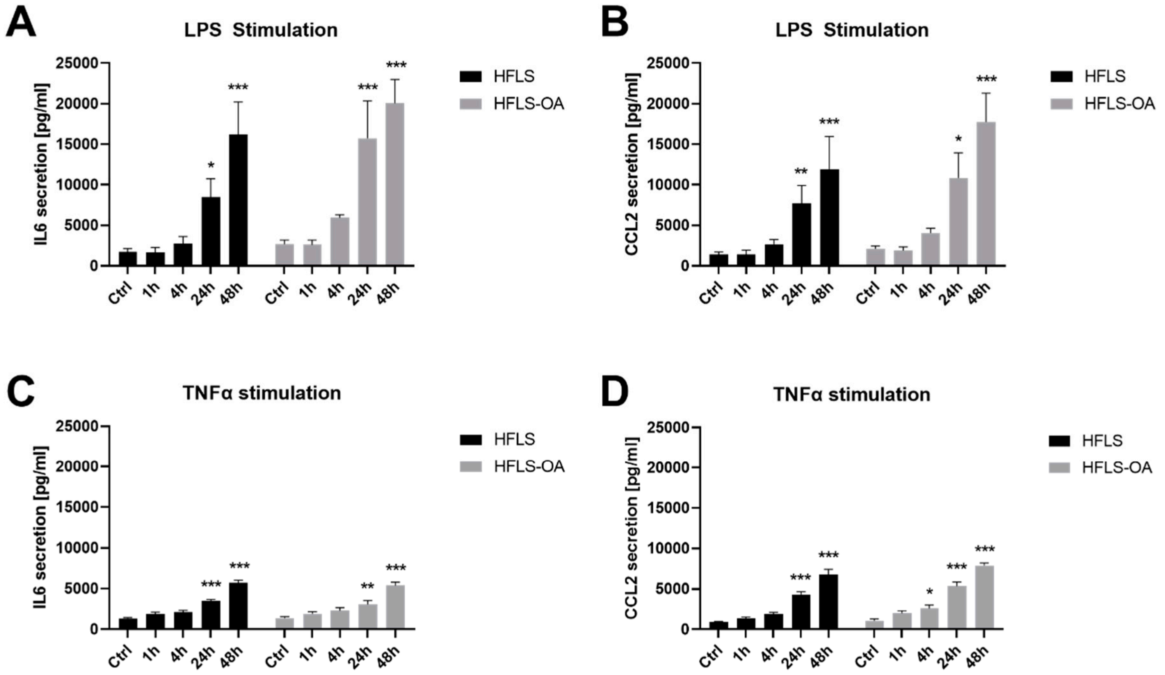

Figure S3.

HFLS and HFLS-OA cells were stimulated with 10 ng/ml LPS (**A**,**B**) or TNFα (**C**,**D**) for different time points (1, 4, 24, and 48 hours). CCL2 or IL6 proteins secretion were measured with the ELISA assays. Data are presented as pg/ml ± SEM and analyzed with one-way ANOVA followed by the Dunnett. * $p < 0.05$; ** $0.01 > p > 0.001$; *** $p < 0.001$ vs. control group.
